# Plant-Based Biosynthesis of Copper/Copper Oxide Nanoparticles: An Update on Their Applications in Biomedicine, Mechanisms, and Toxicity

**DOI:** 10.3390/biom11040564

**Published:** 2021-04-12

**Authors:** Devanthiran Letchumanan, Sophia P. M. Sok, Suriani Ibrahim, Noor Hasima Nagoor, Norhafiza Mohd Arshad

**Affiliations:** 1 Centre for Research in Biotechnology for Agriculture, University of Malaya, Kuala Lumpur 50603, Malaysia; devanthiran@um.edu.my (D.L.); sophiasokpm@gmail.com (S.P.M.S.); hasima@um.edu.my (N.H.N.); 2 Institute of Biological Sciences (Genetics and Molecular Biology), Faculty of Science, University of Malaya, Kuala Lumpur 50603, Malaysia; 3 Department of Mechanical Engineering, Faculty of Engineering, University of Malaya, Kuala Lumpur 50603, Malaysia; sue_83@um.edu.my

**Keywords:** copper/copper oxide, green synthesis, nanoparticles, biomedicine, mechanism, toxicity

## Abstract

Plants are rich in phytoconstituent biomolecules that served as a good source of medicine. More recently, they have been employed in synthesizing metal/metal oxide nanoparticles (NPs) due to their capping and reducing properties. This green synthesis approach is environmentally friendly and allows the production of the desired NPs in different sizes and shapes by manipulating parameters during the synthesis process. The most commonly used metals and oxides are gold (Au), silver (Ag), and copper (Cu). Among these, Cu is a relatively low-cost metal that is more cost-effective than Au and Ag. In this review, we present an overview and current update of plant-mediated Cu/copper oxide (CuO) NPs, including their synthesis, medicinal applications, and mechanisms. Furthermore, the toxic effects of these NPs and their efficacy compared to commercial NPs are reviewed. This review provides an insight into the potential of developing plant-based Cu/CuO NPs as a therapeutic agent for various diseases in the future.

## 1. Introduction 

**Figure 1:**
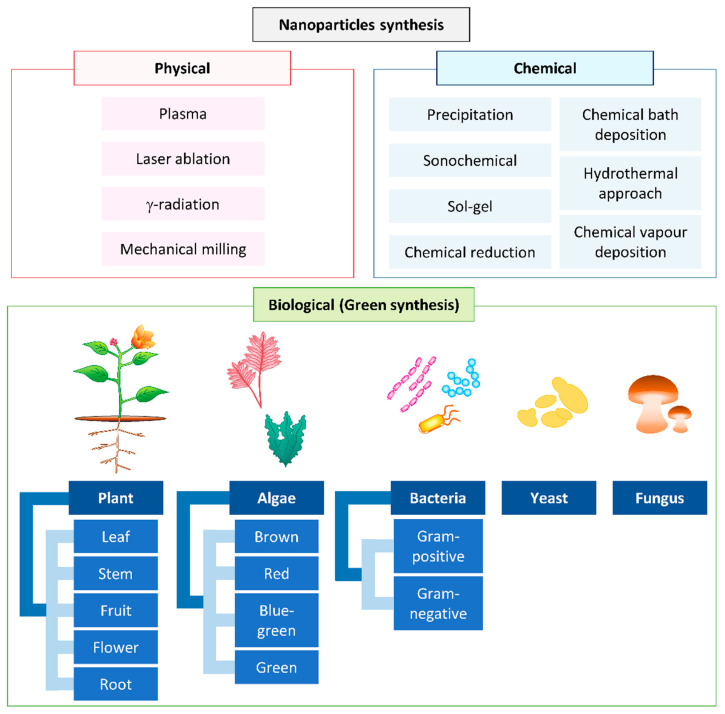
Conventional methods used for nanoparticle synthesis.

Nanoparticles (NPs) are materials ranging in size from 1–100 nm, with unique properties compared to bulk materials [1]. Their higher surface-area-to-volume ratio is a very important unique property that allows them to be used in different fields of chemical, food, electronic, and healthcare industries [2,3,4]. There are various physical, chemical, and biological approaches to synthesizing NPs (Figure 1).

Generally, physical and chemical methods are used commercially for the synthesis of NPs. Chemical methods such as the electrochemical method [5], precipitation [6], the sonochemical route [7], sol-gel [8,9], the hydrothermal approach [10,11], chemical bath deposition [12], chemical reduction [13], and chemical vapor deposition use harsh reducing agents, organic solvents, and toxic chemicals and produce dangerous byproducts [14,15,16]. The use of harsh synthetic chemicals, including sodium borohydride [17], hypophosphite [18], and hydrazine [19], as reducing agents in the chemical approach leads to the adsorption of harsh chemicals on the surface of the synthesized NPs and, eventually, increase their toxicity [20]. On the other hand, the physical methods, such as plasma, pulsed laser, gamma radiation, and mechanical milling, normally require high energy and are more time-consuming to scale-up compared to green synthesis [16,21,22]. 

In recent years, the development of an efficient “green” chemistry method for synthesizing metal NPs has become a major focus of researchers. Green synthesis of Cu and CuO NPs is more advantageous than chemical and physical synthesis as it is a clean, nontoxic, cost-effective, and environmentally friendly approach. It bypasses the use of harsh, toxic, and expensive chemicals [22] and, instead, utilizes biological entities like bacteria [23], yeasts [24], fungi [25], algae [26], and plants [20]. Among all these natural organisms used in the green synthesis of Cu and CuO NPs, plants rich in bioactive compounds can serve as a reducing, stabilizing, and capping agent during NP synthesis; this makes them the best choice. They are nonpathogenic to humans, and the downstream processing steps are simple [27]. Unlike some bacterial and fungal strains that produce the NPs intracellularly, plant-mediated NP synthesis yields the NPs in the mixture solution, which can be easily obtained by filtering, rinsing, and drying [11,28]. The size, morphology, and stability of NPs can also be easily optimized for medicinal and pharmaceutical usage using this green method [5,29,30]. In this review, we focus on the updates of Cu and CuO NPs synthesized from plants and their medicinal application. 

## 2. Copper Nanoparticles 

In recent years, the development of metal and metal oxide NPs has greatly enhanced the biomedical field in terms of biosensing, imaging, diagnosis, and therapy [31,32,33,34]. The most commonly used metals and their oxides are gold (Au), silver (Ag), and copper (Cu). Among these, Cu is a relatively low-cost metal that is more cost-effective than Au and Ag [35]. Additionally, Cu NPs are efficient catalysts, with high yields and easy product separation, and they can be reused repeatedly [36].

In fact, Cu is needed in a minimal amount, together with the essential enzymes in the human body. For example, superoxide dismutase, cytochrome oxidase, and tyrosinase are made up of this trace element [37]. However, Cu-free ions are potentially harmful to the human body at the cell, organ, and body levels. Therefore, Cu ions in living organisms should be regulated [38].

Cu NPs can be easily oxidized to form copper oxides (CuOs), which are inorganic NPs. Both Cu and CuO NPs are used extensively as anticancer, antimicrobial, and antioxidant agents [39,40]. This is mainly due to the fact that NPs are able to interact with the biological system at cellular levels for various reactions and functions [41,42].

## 3. Plant-Based Green Synthesis of Copper and Copper Oxide Nanoparticles 

Plants produce numerous secondary metabolites and contain phytochemicals, which are potential bioresources for synthesizing Cu and CuO NPs. The most important phytochemicals in plants are phenols and flavonoids, found in different parts of the plants, such as shoots, leaves, stems, flowers, roots, and fruits. These phenolic compounds possess hydroxyl and ketone groups, contributing to the iron chelation and subsequently demonstrating a strong antioxidant property [43]. NPs synthesized through this green method increased in stability, prevented the agglomeration and deformation of the NPs and allowed adsorption of phytochemicals on the surface of the NPs, which enhance the reaction rate of NPs [44]. 

**Figure 2:**
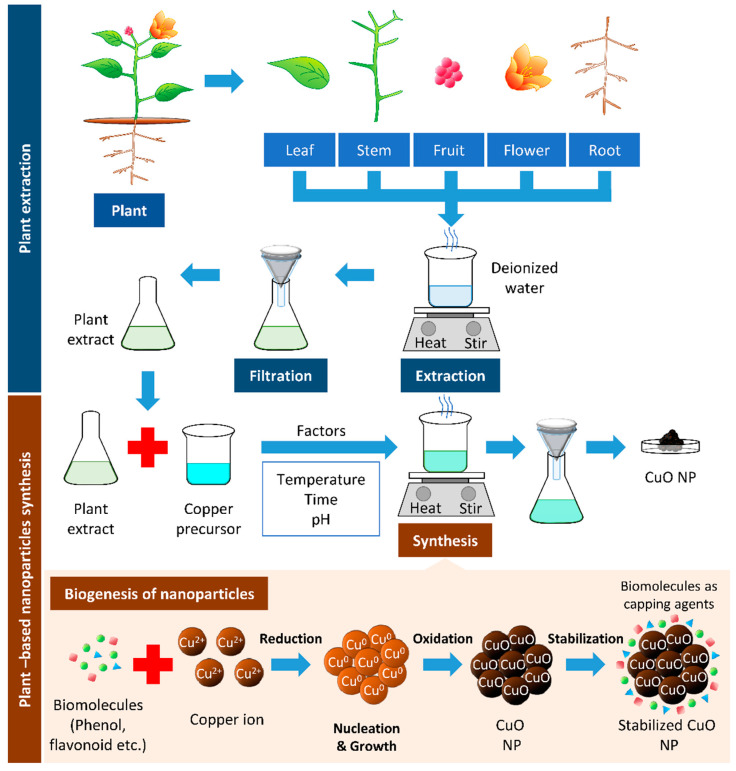
Plant-based synthesis of copper oxide nanoparticles. The boiling of respective parts of the plant can be used to extract biomolecules from the plant. The resulted filtrate is the plant extract, which can reduce the copper precursor to synthesize copper/copper oxide nanoparticles (Cu/CuO NPs). The biomolecules’ presence in plant extracts serves as reducing, stabilizing, and capping agents in Cu/CuO NP synthesis.

One of the common techniques in synthesizing Cu and CuO NPs is mixing a known concentration of plant extract to a known concentration of precursor and heating the mixture to a fixed temperature, with continuous stirring at a given time (Figure 2, Table 1).

**Figure 3:**
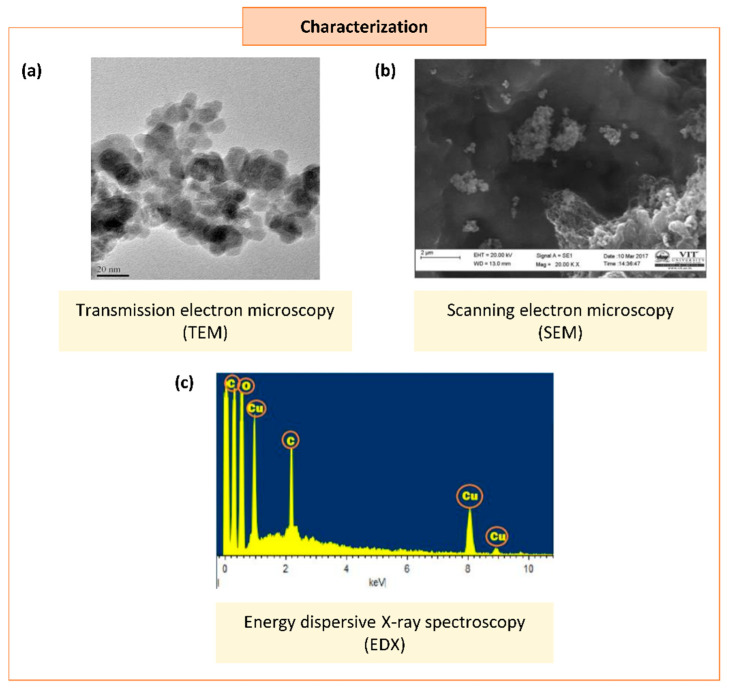
Characterization of synthesized copper/copper oxide nanoparticles (Cu/CuO NPs). (**a**) Transmission microscopy image of CuO NPs synthesized from *Syzygium alternifolium* (Wt.) Walp [84]. (**b**,**c**) Scanning electron microscopy image (SEM) (**b**) and energy dispersive X-ray spectroscopy EDS (**c**) of Cu NPs synthesized using *Cissus vitiginea* leaf extract [62].

Occasionally, certain organic chemicals were added to prevent agglomeration and obtain fine NPs while maintaining the greener approach. The synthesized NPs eventually form various shapes and sizes, which carry unique properties in many applications [20]. Different characterization techniques can analyze the physical and chemical properties of NPs. In addition to size and shape, the size distribution, degree of aggregation, surface charge, surface area, and surface chemistry of the NPs can also be analyzed [88] (Figure 3).

The first step of characterization study after the synthesis of NPs is the crystal structure and its chemical composition [89]. The analytical tools of scanning electron microscopy (SEM), tunneling electron microscopy (TEM), dynamic light scattering (DLS), particle analyzers, and field emission scanning electron microscopy (FESEM) were used to analyze the size and morphology of the Cu/CuO NPs., while UV–visible spectroscopy (UV–vis), X-ray diffraction (XRD), Fourier transform infrared spectroscopy (FTIR), surface plasmon resonance, and energy dispersive X-ray spectroscopy (EDS) were used to analyze the elemental chemical compositions of Cu/CuO NPs [90]. Based on our literature search, the size of Cu/CuO NPs synthesized using a plant source resulted in sizes ranging from 2–577 nm, with spherical, oval-shaped, plate, rectangular, cuboidal, and cubical shapes (Table 1). 

### 3.1. Synthesis Strategy of CuO, Cu_2_O, and Cu_4_O_3_ NPs

Cu exists with variable oxidation numbers; this includes Cu(I), Cu(II), and very few Cu(III) ions. To date, the synthesis strategy reported for CuO, Cu_2_O, and Cu_4_O_3_ is the same in terms of parameters such as plant extract, precursor concentration, pH, and temperature. However, it is these parameters that mainly affect the type of Cu particles formed during green synthesis [91]. The synthesized CuO, Cu_2_O, and Cu_4_O_3_ NPs can be differentiated by observing the position of the absorption peaks in the UV–vis spectra analysis. The peak width indicates the size distribution, and the peak height indicates the number of Cu particles produced. At optimum synthesis parameters, a high absorption peak at around 560–585 nm implies a high yield of Cu particles [92]. This is supported by a study conducted by Sampaio et al. (2021) to differentiate between Cu and Cu_2_O through green synthesis using *Cynara scolymus* L. The authors reported that a UV–vis spectra absorbance peak at 386 nm indicates the presence of Cu_2_O, while an absorbance peak at 565 nm detects the presence of Cu particles. Additionally, further differentiation is also done using other analyses, such as EDS spectrum analysis to show elemental compositions and SEM for size distribution (Cu_2_O NPs with Cu of 81.34% and sizes of 324–711 nm; Cu NPs with Cu of 73.77% and sizes of 364–1125 nm) [93]. The absorbance peak of Cu_4_O_3_ synthesized using *Razma* seed under UV–vis spectra was 270–372 nm [94], which was much lower than Cu and Cu_2_O, as reported by Sampaio et al. (2021). 

### 3.2. Factors Affecting the Green Synthesis of Cu and CuO NPs

A few parameters play a crucial role in determining the characteristics of the Cu and CuO NPs synthesized; these include reaction temperature, pH, time, concentration of plant extracts, precursor used, and mixing speed (Table 1). In fact, it is the multiple parameters that affect the synthesis of NPs with the desired size and morphology. Therefore, monitoring these parameters could produce NPs with unique properties and promising applications that are easy to scale up with a shorter synthesis time [95]. 

#### 3.2.1. Temperature 

The reaction medium’s temperature was found to influence the nature of the NPs formed. As the reaction temperature increases, the reaction rate increases, consuming metal ions to form the nuclei of the NPs; this may lead to smaller NP sizes [96]. For instance, the biosynthesis of NPs using *Ixiro coccinea* leaf extract at 27 °C for 24 h produces NPs with a bigger size (80–110 nm) than the NPs (10–50 nm) produced using *Pterolobium hexapetalum* leaf extract at 60 °C for 2 h using the same precursor [72,81]. These findings suggest that an elevation in temperature reduces the length of time of synthesis, independent of the source of plant extract used. 

Generally, green synthesis is conducted at below 100 °C or at ambient temperature [97]. This is because the biomolecules and active components found in the plant extract can easily degrade at high temperatures [98]. For example, at 27 °C, the CuO NPs synthesized from *Fragaria ananassa* fruit extract [71] and *Cissus vitiginea* leaf extract [62] yielded sizes of NPs ranging from 10–30 nm and 5–20 nm, respectively. On the other hand, *Camellia sinensis* leaf extract produced NPs of 67–99 nm at 95 °C [60]. These studies suggest that room temperature is favorable for synthesizing small-sized NPs. 

In contrast, a study conducted by Sulaiman et al. (2018) using *Olea europaea* leaf extract at high temperature, such as 100 °C, produced NPs with relatively small size (20–50 nm) [78]. In support of this, a recent study unveiled that the green synthesis of CuO NPs using *Eucalyptus globulus* leaf extract decreased in size from 68 to 12 nm when the temperature of the reaction mixture was elevated from 30 to 140 °C [66]. These controversial findings may be due to the effect of other parameters such as phytochemical composition of the plant, pH, time of reaction, and precursor used. 

In some studies, different aspects of temperature were focused on, namely, the calcination and annealing of CuO NPs. Calcination and annealing are known as heat treatments performed postsynthesis to decompose and purify a material. It was demonstrated that the size of synthesized NPs is proportional to annealing and calcination temperatures. This is evident in the research conducted by Fardood and colleagues using black tea extract, whereby the size of the CuO NPs increased from 22.3 to 38.7 nm when the annealing temperature increased from 500 to 800 °C, respectively [59]. Similarly, the size of henna-extract-mediated CuO NPs increased from 22.5 to 38.3 nm when the annealing temperature increased from 400 to 800 °C [73]. A recent comparative study reflected that a reduction in the size of annealed CuO NPs is due to the difference in phase transitions between unannealed and annealed NPs [67]. The annealing process also resulted in black precipitate agglomerated and highly characterized cubic-shaped NPs compared to the elongated morphology obtained from unannealed NPs [67]. These data indicate the implication of temperature on the size of Cu/CuO NPs.

#### 3.2.2. Time

NPs can undergo aggregation, shrink, or grow during long-term storage and it also has a shelf life that eventually affects its overall potential [99,100]. A study by Mudunkotuwa et al. (2012), comparing new and aged Cu NPs, reported a change in size and also chemical behavior of the Cu NPs. The size of new Cu NPs increased from 10 ± 5 to 18 ± 10 nm for Cu NPs aged under ambient conditions. It was also found that the aged Cu NPs were highly oxidized compared to new Cu NPs [101]. A similar trend was also observed in the duration of the green synthesis reaction performed. Studies have implied that the size of NPs is dependent on the reaction time [49,64,73,87]. It can be seen that increases in the time taken for green synthesis increase the size of the NPs. From Table 1, green synthesis of Cu and CuO NPs using *Allium eriophyllum* Boiss at 16 h produced NPs with a larger size (30–35 nm), while synthesis using *Ailanthus altissima* at 4 h produced smaller sized (5–20 nm) NPs [47,49]. However, time does not greatly influence the size of the NPs, as other factors such as type of plant extract, precursor, and temperature, have even more effect, as evident in Table 1. 

#### 3.2.3. Concentration of Plant Extracts 

Plants with bioaccumulation and heavy-metal detoxification properties are the best for NP synthesis [20]. The biomolecules in plant extracts can act as natural capping and reducing agents during the green synthesis of NPs. These metabolite compositions vary depending on the types and parts of the plant and the extraction procedure [102]. Hence, the volume of plant extracts and the amount of biomolecules present in the plant extract used can affect the synthesis rate due to the availability of these molecules for the rapid bioreduction of metal salts and the stabilization of NPs [103]. It was demonstrated that increased concentrations of plant extract speed up the rate of reduction of copper ions in the solution, which directly increases the formation rate of Cu and CuO NPs [80]. This is attributed to the phytochemicals present in the plant extract, which are responsible for the bioreduction and stabilization of the NPs [80].

In addition to speeding up the reaction, an increase in the concentration of plant extract also decreases the size and alters the shape of the CuO NPs formed. A recent study demonstrated that synthesizing CuO NPs using a different volume of sugarcane juice, ranging from 2–10 mL, produced CuO NPs that were 29.89–22.80 nm in size [82]. Notably, the CuO NPs synthesized were spherical when using a higher concentration of sugarcane juices. In contrast, various shapes were observed from the CuO NPs synthesized with lower concentrations of sugarcane juice, ranging from spherical, square, cube, and plate shapes to a rectangular shape, along with some irregular shapes [82]. This is supported by previous studies demonstrating that increasing the concentration of plant extract when synthesizing NPs alters NP morphology, from irregular to spherical, and the size of the NPs [80]. These studies provide evidence on the interchange of morphology based on the plant extract used. 

#### 3.2.4. Precursor Used

The precursor is a salt and alkali metal solution. Numerous soluble copper salts were used in the green synthesis of Cu and CuO NPs, such as copper chloride (CuCl_2_), copper sulfate (CuSO_4_), copper acetate (Cu(OAc)_2_), and copper nitrate (Cu(NO_3_)_2_). Copper salts are the primary source for releasing Cu^2+^ ions in reaction mixtures; the bioreduction of these ions by the plant extracts forms the complete NP [80]. 

There is limited information for comparison between the different precursors used and their effect on the green synthesis of Cu and CuO NPs. A comparative study between different precursors such as CuCl_2_, Cu(NO_3_)_2_, and CuSO_4_, used in the physical synthesis of CuO NPs by Sanjini et al. (2017), reported that changing the precursor has no significant impact on the crystal structures of CuO NPs. In this study, the synthesis of CuO NPs using the precursor CuCl_2_ formed CuO NPs of 31 nm, 34 nm when using Cu(NO_3_)_2_ and 56 nm when using CuSO_4_. Hence, this indicated that CuO NPs synthesized using CuCl_2_ as a precursor may produce smaller NPs [104]. However, another study conducted by Phiwdang et al. (2013), using precursors CuCl_2_ and Cu(NO_3_)_2_, reported that CuCl_2_ forms spherical-shaped particles, while Cu(NO_3_)_2_ forms rod-shaped particles. The result also suggested that better formation and good crystallinity of CuO NPs were obtained using the Cu(NO_3_)_2_ precursor compared to CuCl_2_ [105]. It was also revealed that a low concentration of the precursor produced smaller-sized NPs, whereas a high concentration of the precursor produced larger NPs with faster agglomeration [95,106]. The results from these different studies suggest that in-depth experimental work is needed to determine the effect of precursors on the size and shape of NPs synthesized from plants. 

#### 3.2.5. pH

The pH of the reacting mixture in the biosynthesis of NPs is also one of the factors affecting the size of NPs. The optimum pH of the reaction medium ranges from 7–9, suggesting an alkaline condition is favorable for synthesizing NPs [107]. As evident from Table 1, biosynthesis of NPs occurring at the alkaline condition of pH > 8 produces smaller-sized NPs. The NPs synthesized in an acidic condition of pH < 7 were relatively larger, up to 153.7 nm [67]. A study conducted by Rajesh and his co-researchers has provided evidence that when the pH of the reaction mixture increased from 6 to 10, the Cu NP size decreased from 32 to 20 nm [108]. However, the influence of pH on the morphology of CuO NPs was reported by Singh et al. (2019). The synthesis of CuO NPs at pH 10 (alkaline) produces loosely agglomerated NPs, with sharp CuO NPs with a particle size of 28.2 nm. In contrast, more agglomerated CuO NPs, with a size of 66.3 nm, were produced at pH 7 (neutral). Interestingly, the CuO NPs synthesized at pH 10 showed higher genotoxicity than CuO NPs at pH 7 [109]. The evidence given is based on chemical synthesis; limited study on green synthesis has been conducted so far for pH. 

## 4. Application of Cu and CuO NPs 

In this modern era, NPs have been widely employed in cosmetics, textiles, photonics, agriculture, and heavy industries as catalysts. Additionally, the application of NPs has been extended to medicine. Available data suggest that Cu and CuO NPs can be used as antimicrobial, antifungal, anticancer, and anti-inflammatory agents and therapeutic agents for wound healing. The detail on these, including the mechanism, will be discussed in the following sections. 

### 4.1. Antibacterial 

Studies have showed that the Cu/CuO NPs have inhibitory growth effects against Gram-negative and Gram-positive bacteria (Table 2).

Plant-mediated Cu NPs effectively repressed Gram-negative bacteria in urinary tract infection, such as *Escherichia coli* (*E. coli*), *Enterococcus* sp., *Proteus* sp., and *Klebsiella* sp. [62]. This implies the potential of these NPs in treating such infection. The plant-derived NPs can also inhibit *K. pneumonia*, which is pathogenic if spread to other parts of the body [51].

Cu and CuO NPs have also shown a suppressive effect on the growth of Gram-positive bacteria *Staphylococcus aureus* (*S. aureus*), *Bacillus subtilis* (*B. subtilis*), and *Streptococcus pyrogenes* (*S. pyrogenes*). *S. aureus* is a pathogen known to cause respiratory and skin infections, while *B. subtilis* can cause diarrhea. In contrast, *S. pyrogenes* infects humans, causing various infections such as rheumatic fever and scarlet fever [51]. 

It was revealed that sugarcane-juice-mediated NPs are resistant to *E. coli* compared to Gram-positive bacteria *B. subtilis* and *S. aureus* [82]. However, a comparative study between Gram-negative bacteria, *E. coli* and *P. aeruginosa*, and gram-positive bacteria, *S. aureus* and *B. cereus*, with the treatment of CuO NPs synthesized using fruit extract *Tribulus terrestris* reported that the minimal inhibitory concentration (MIC) is higher in Gram-positive bacteria compared to Gram-negative bacteria [81,86]. Altogether, it suggested that diverse size and characteristics of Cu/CuO NPs are required to treat various pathogenic infections based on the type of bacterial agent involved. 

#### Predicted Mechanism for Antibacterial Activity

**Figure 4:**
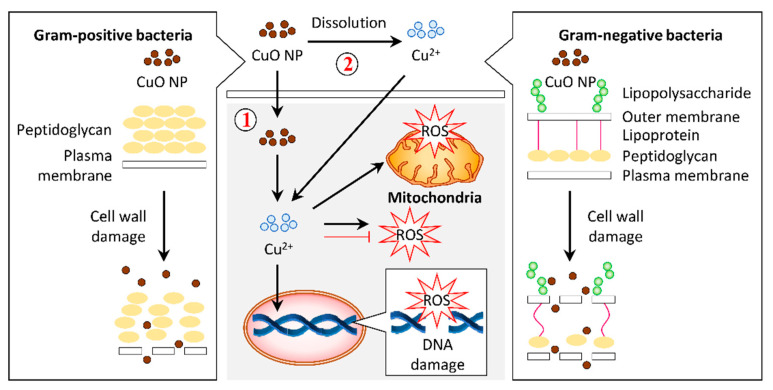
Graphical representation of the proposed mechanism for antibacterial activity in response to copper oxide nanoparticles (CuO NPs). (1) Gram-positive and Gram-negative bacteria have different cell wall compositions, which can be destroyed by CuO NPs. This cell wall damage results in bacteria lysis. Internalization of CuO NPs eventually releases Cu^2+^ ions in the cytosol, leading to reactive oxygen species (ROS) accumulation in the bacteria and, subsequently, DNA and mitochondria damage. Alternatively, CuO NPs may release Cu^2+^ ions and permeate into the cells to stimulate the cellular response that contributes to bactericidal activity.

Generally, the treatment of Cu and CuO NPs permeates the Cu^2+^ ions intracellularly by interacting with the bacterial cell membrane [64]. Distortion of the bacterial cell membrane was observed under FESEM in response to CuO NPs synthesized from aqueous dried fruit extract of *T. terrestris* [86]. These CuO NPs also induced ROS generation that accounted for the antibacterial activity (Figure 4) [86].

Many plant-based Cu/CuO NPs that demonstrated an antibacterial effect tend to have antioxidant properties [49,51]. It was found that *C. vitiginea*-mediated Cu NPs demonstrate antioxidant activity, which contributes to growth inhibition of urinary tract infection pathogens [62]. Likewise, the CuO/Cu NPs from *Allium sativum* extract and *Allium eriophyllum* Boiss leaf extract exhibit antibacterial activities that may be due to their antioxidant property [49,51]. 

### 4.2. Antifungal

Fungi can cause infections to humans through direct contact. *A. flavus* is a pathogenic fungus that causes liver cancer in humans when spoiled food is consumed. *A. fumigates* produces airborne spores, which can cause chronic lung disease if inhaled. *C. albicans*, found in our body at the mouth and vagina, can cause infections if there is overgrowth [51]. Cu/CuO NPs exerted antifungal properties, suggesting their potential in treating fungal infection (Table 3).

**Figure 5:**
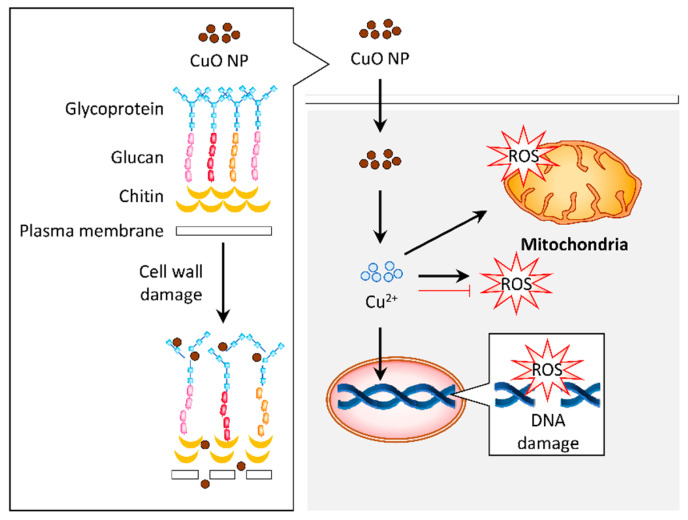
Graphical representation of the proposed mechanism for antifungal activity in response to copper oxide nanoparticles (CuO NPs). The CuO NPs distorted the cell wall of fungus such as *Aspergillus flavus*. Internalization of these particles induced reactive oxygen species (ROS) generation, which resulted in DNA and mitochondrial damage that contribute to the antifungal activity. Alternatively, the antifungal activity of CuO NPs may be attributed to their antioxidant property.

A study has highlighted that the Cu NPs synthesized using *A. eriophyllum* Boiss leaf extract is more resistant against fungi such as *C. guilliermondii* and *C. krusei* compared to bacteria [49]. The fungus has numerous layers of lipids in its cell wall, which makes it challenging for the entry of NPs into the organism [110]. A recent study noted that the green synthesis of CuO NPs triggered damage to the cell wall and accumulated ROS in *A. flavus* to demonstrate an antifungal effect [66]. In addition, the CuO NPs synthesized from *A. sativum* extract also exhibited an antioxidant activity that may contribute to the antifungal property [49,51]. The limited data implies that the CuO NPs can induce antifungal properties via different mechanisms (Figure 5).

### 4.3. Anticancer

Numerous studies have reported the anticancer effect of plant-mediated Cu/CuO NPs, particularly breast [37,53,54,66,78,81,111,112], cervical [53,54,79], colon [37], skin (epithelioma) [54], gastric [86], blood (leukemia) [37], liver [65], lung [54,56,69,70] and ovarian [78] cancers (Table 4). 

To determine the anticancer effect of Cu/CuO NPs, the cancer cells, maintained at 37 °C with a continuous supply of 5% carbon dioxide in their respective medium, were seeded in a 96-well plate prior to NP treatment [113,114]. There are various colorimetric assays used in the study of toxicity of cancer cells, such as MTT (3-(4,5-dimethylthiazol-2-yl)-2,5-diphenyl tetrazolium bromide), MTS (3-(4,5-dimethylthiazol-2-yl)-5-(3-carboxymethoxyphenyl)-2-(4-sulfophenyl)-2H-tetrazolium), and WST (4-[3-(4-Iodophenyl)-2-(4-nitro-phenyl)-2H-5-tetrazolio]-1,3-benzenesulfonate). These assays measure metabolic activity in viable cells based on the reductase activity of the mitochondria [115].

The toxicity to cancer cells varies with different sizes and shapes of the NPs. Previous studies have proposed that large-sized Cu NPs have a higher half-maximal inhibitory concentration (IC_50_) value than small-sized NPs in A549 lung cancer cells and MCF-7 breast cancer [52,54]. Paradoxically, studies also revealed that NPs with size >200 nm have a lower IC_50_ on MCF-7 and MDA-MB-231 breast cancer cells compared to the 5–24 nm NPs [66,111]. 

Notably, these Cu/CuO NPs were synthesized using a different source of plants under different conditions. A cytotoxic study conducted by Rehana et al. (2017) on various cancer cells (MCF-7, HeLa, Hep-2, and A549) treated with CuO NPs synthesized using different types of plant extracts from *Azadirachta indica, Hibiscus rosa-sinensis, Murraya koenigii, Moringa oleifera,* and *Tamarindus indica* demonstrated different levels of cytotoxic effects. Among these, CuO NPs synthesized using *T. indica* leaf extract exerted higher cytotoxicity against all cancer cells, as evidenced by the lower IC_50_ [54] (Table 4). It was found that the *T. indica* leaf extract contains more proteins, amino acids, carbohydrates, higher flavonoids, glycosides, phenolic compounds, saponins, and tannins compared to other plant extracts [54]. This may explain the difference in the anticancer activity of the NPs of similar size.

#### Predicted Mechanism for Anticancer Activity 

**Figure 6:**
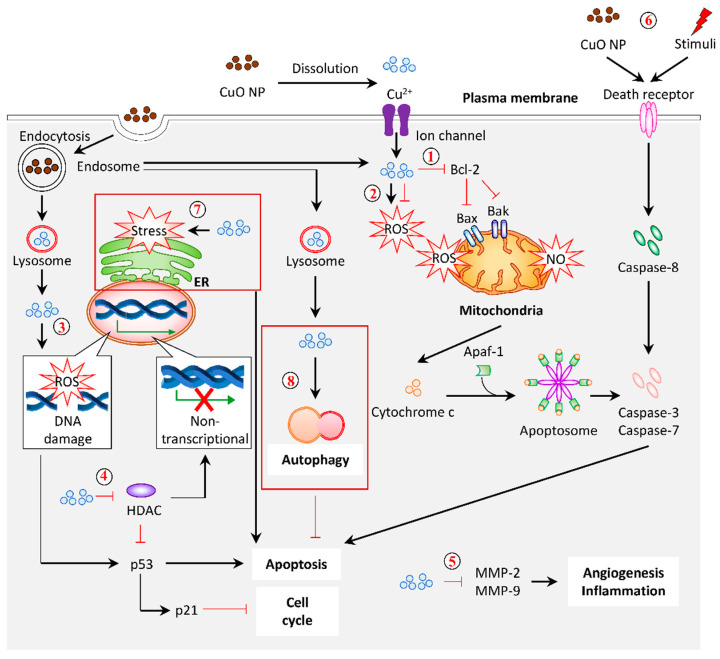
Graphical representation of the proposed mechanism for anticancer activity in response to CuO NPs (referring to plant-based NPs, unless stated otherwise). (**1**) Mitochondrial damage and intrinsic apoptosis. CuO NPs from plants suppressed Bcl-2 (antiapoptotic protein). They promoted Bak/Bax (proapoptotic protein) expression to induce mitochondria membrane permeabilization, followed by the release of cytochrome c. This cytochrome c, together with the Apaf-1 adaptor and pro-caspase-9, forms apoptosome. The activated caspase-9 triggers apoptosis via caspase-3 and caspase-7. (**2**) Antioxidation/ROS generation. Anticancer effects of CuO NPs often demonstrate an antioxidant property. Conversely, the elevation of intracellular ROS levels in response to CuO NP treatment was reported. (**3**) DNA damage. The CuO-NP-inducible intracellular ROS generation rendered DNA damage, which upregulated the expression of p53 and p21, which is responsible for apoptosis and cell cycle arrest. (**4**) Histone deacetylases (HDACs). The treatment of CuO NPs decreased the total amount of histone deacetylases, which may account for the increased mRNA and protein levels of p53 and p21. (**5**) Oncogenes. The mRNA and protein levels of MMP-2 and MMP-9 were downregulated in response to CuO NPs, which may protect against angiogenesis and inflammation. (**6**) Extrinsic apoptosis. Alternatively, CuO NPs and other stimuli can activate the extrinsic apoptosis by interacting with the death receptor on the cell membrane to recruit caspase-8. The activated caspase-8 processed caspase-3 and caspase-7 to induce apoptosis. (**7**) ER-stress-induced apoptosis. Commercial CuO NPs initiated apoptosis via ER-stress-associated caspase-12. (**8**) Autophagy. Commercial CuO NPs induced autophagy with a high expression of LC3-II and ATG5, acting as a defense response against the treatment. Dark red box indicates the predicted mechanism based on the evidence of commercial CuO NPs.

A plethora of studies has been reported on the application of plant-based Cu/CuO NPs. However, the precise mechanism or signaling in regulating the activity of these NPs remained ambiguous. Based on the available data of plant-based and commercial Cu/CuO NPs, we summarize the mechanism of these NPs, demonstrating their effects (Figure 6).

The generation of reactive oxygen species (ROS) is known to be the major contributor to cell death induced by Cu/CuO NPs synthesized from plants (Figure 6 (**1**)). Mitochondria are the center of energy metabolism and cellular signaling [116]. The disruption of this organelle is implicated in the accumulation of reactive oxygen species (ROS) and nitric oxide (NO), and, subsequently, oxidative stress [117]. Alteration of the mitochondria structure, accompanied by the loss of membrane potential, was observed in response to black-bean-extract-mediated CuO NPs in HeLa cervical cancer cells, signifying mitochondrial damage [79]. Moreover, the intracellular ROS level was significantly increased in response to CuO NPs synthesized from black bean extract and leaf extract of *Ficus religiose*, *E. globulus*, and *P. hexapetalum* [66,69,79,81]. It was accompanied by the loss of mitochondrial membrane potential in A549 lung cancer, HeLa cervical cancer cells, and MCF-7 breast cancer cells, respectively [66,69,79]. In addition to the accumulation of ROS, the uptake of CuO NPs synthesized from *A. indica* leaf also upregulated the NO level in MCF-7 and HeLa cancer cells [53].

On the other hand, studies have suggested that an increased ROS level may be detected in cancer cells due to high metabolic activities. Therefore, suppressing the ROS level is a beneficial approach in this aspect. The CuO NPs produced from *Matricaria chamomilla* flower extract, leaves of *A. indica*, *H. rosa-sinensis*, *M. koenigii*, *M. oleifera*, *T. indica*, *Eclipta prostrate*, and *O. europaea* exhibited an antioxidant property, as shown by their free radical scavenging ability [54,65,78,118] (Figure 6 (**2**)). A recent review proposed that the level of ROS was slightly enhanced in cancer cells, which, in turn, favor them to develop antioxidant mechanisms that enhanced their sensitivity towards external stimuli [116]. This characteristic promotes the production of ROS [116]. Given that the ROS is mandatory in cancer initiation, depletion of the ROS level would be beneficial to overcome cellular transformation [116]. Therefore, it is important to evaluate the role of ROS in disease conditions to select a CuO with a suitable property. 

Excessive oxidative stress in cells leads to cellular damage and cell death. A high intracellular ROS level can trigger DNA damage, which is characterized by DNA fragmentation [116] (Figure 6 (**3**)). The oxidative stress induced by *A. indica* leaf CuO NPs in cancer cells is attributed to DNA fragmentation [53]. This finding is consistent with a recent study that examined the genotoxicity of commercial CuO NPs [119]. In contrast, the CuO NPs produced from *M. chamomilla* flower extract, with antioxidant properties, were found to interact directly with the plasmid, implicated in DNA cleavage [118]. DNA damage is coupled to the expression of tumor suppressor genes (p53 and p21), whereby the upregulation of these genes halts the cell cycle and induces apoptosis [120]. The exposure of NPs promotes DNA damage that eventually halts the cell cycle. Biofabrication of CuO NPs using *E. globulus* leaf extract and *Beta vulgaris* aqueous extract induced cell cycle arrest at the G2/M phase in MCF-7 breast cancer cells and A549 cancer cells, respectively [56,66]. Additionally, the expression of p53 was upregulated [66]. A study proposed that the increased level of tumor suppressor genes (p21 and p53) and the decreased level of oncogenes, MMP-2, and MMP-9 expressions, in response to plant-based CuO NPs, are regulated by the various histone deacetylase (HDAC), enzymes that create a nontranscriptional compact chromatin structure by the removal of the acetyl group on histones [70] (Figure 6 (**5**)).

Apoptosis is a programmed cell death mechanism governed by a series of caspases in response to cellular stress and DNA damage [121]. This cell death can be induced via intrinsic and extrinsic pathways. The intrinsic pathway involves the mediation of proapoptotic proteins, Bak/Bax expression on mitochondria by an antiapoptotic protein, Bcl-2 to induce membrane permeabilization, and the release of cytochrome c to form the apoptosome with the Apaf-1 adaptor and pro-caspase-9 [88]. As for the extrinsic apoptotic pathway, caspase-8 is activated by the ligand and death receptor interaction on the cell surface. Both the activation of intrinsic and extrinsic apoptosis will cleave the major executioner caspases, such as caspase-3 and caspase-7 [121]. Studies have shown that the expression of intrinsic apoptotic mRNA and protein, such as Bax, cytochrome c, caspase-9, and caspase-7, are remarkably upregulated when treated with CuO NPs [53,66,70] (Figure 6 (**1**)). The expression of caspase-8 also increased simultaneously in the CuO-NPs-treated cells, signifying the potential to initiate extrinsic apoptosis [53,70] (Figure 6 (**6**)). It seems that the plant-mediated CuO NPs induced apoptosis via both intrinsic and extrinsic pathways.

Recently, it was reported that commercial CuO-NP-induced apoptosis could be triggered by endoplasmic reticulum (ER) stress, as evidenced by the upregulation of ER-stress-related mRNA and protein expression, implicating the multiple pathways involved in inducing apoptosis [122] (Figure 6 (**7**)). A previous study employed the lipidomics approach to reveal the alteration in lipid profile upon commercial CuO NP exposure in the HCT-116 colon cancer cell line [123]. Interestingly, the CuO NP treatment promotes autophagy but not apoptosis, which may contribute to cancer cell death [123] (Figure 6 (**8**)). In fact, this autophagy induction may act as a cellular defense against the toxicity of CuO NPs, whereby the inhibition of autophagy, either pharmacologically or genetically, successfully triggers apoptosis in CuO-NP-treated cells [124].

Taken altogether, the CuO NPs have demonstrated their anticancer effects via multiple signaling routes, such as ROS generation, antioxidant property, cell cycle arrest, apoptosis, and autophagy. The mode of action of CuO NPs may vary depending on the route and source of Cu/CuO NP synthesis and the cell line used. Current studies of NPs focus on the application and effects, but not the molecular mechanism or signaling. For example, it was documented that CuO NPs from sinapic acid from plants have an antiangiogenic effect on human breast cancers, but their molecular pathway remains unknown [111]. It is crucial to elucidate the precise molecular mechanism of these NPs in different conditions and cell types to provide a better insight into their application in biomedicine.

### 4.4. Wound Healing and Anti-Inflammatory Activity

In addition to antibacterial, antifungal, and anticancer effects, plant-mediated Cu/CuO NPs also demonstrate wound healing and anti-inflammatory properties. The biosynthesis of Cu NP ointment from plants such as *F. ananassa* (Strawberry) and *A. eriophyllum* has wound-healing effects, as shown by reduced neutrophil and lymphocyte recruitment as well as cutaneous wound closure in rats [49,71]. In the mouse model of inflammatory pain induced by various stimuli, *Abies spectabilis*-mediated CuO NP treatment significantly suppressed the infiltration of leukocytes and inflammatory cytokines [45]. The potency of *A. sativum* CuO NPs in inhibiting the inflammation triggered by egg albumin has been documented [67]. These studies indicate the potential of plant-mediated Cu/CuO NPs in reducing inflammation, relieving pain, and promoting wound healing.

In contrast, a recent study using macrophages has reported that commercial CuO NPs and their ions can activate the NLRP3 inflammasome, an intracellular sensor that regulates the release of proinflammatory cytokines [125]. This finding suggests that phytochemicals that served as capping agents in plant-mediated CuO NPs can suppress the inflammatory response.

## 5. Toxicity Evaluation

Although Cu/CuO NPs have various therapeutic effects, the toxicity of these NPs against normal cells and vital organs in humans can cause undesired adverse effects. This aspect should be evaluated thoroughly before utilizing these NPs in medicine. Here, we review the toxicity of plant-based green-synthesized Cu and CuO NPs in vitro and in vivo studies.

A previous study has demonstrated that plant-mediated Cu NPs are relatively safe on normal human cell lines (Table 5).

It has been shown that normal human umbilical vein endothelial cells (HUVECs), treated with up to 1000 μg/mL of Cu NPs biosynthesized from strawberry, remain highly viable [49]. The IC_50_ values reported for the treatment with Cu and CuO NPs on normal cell lines are higher than in cancer cells, notably on HEK 293 cells with IC_50_ 410 μg/mL and normal human dermal fibroblast (NHDF)/L929 cells with IC_50_ > 100 μg/mL. Although the IC_50_ value of CuO NPs in HuFb (human dermal fibroblast) was 53.34 μg/mL, these NPs have IC_50_ values of <2.5 μg/mL in breast and ovarian cancer cell lines [78] (Table 4 and Table 5). These data revealed the Cu and CuO NPs have minimal toxicity in normal cells, with a cytotoxicity effect on cancer cells at low concentrations, suggesting their safe application.

Nevertheless, the toxicity and optimal dosage to be administrated should be evaluated. It has been shown that the oral administration of green-synthesized Cu NPs to male Swiss albino mice may affect the digestive system, which contributed to a reduction in the weight of the spleen, with an elevated weight of the liver and kidneys in a dose-dependent manner [78]. In this study, no significant toxicity was observed up to 400 mg/kg, but it was lethal at 800 mg/kg [78]. However, a study conducted in zebrafish embryos revealed that the plant-synthesized CuO NPs tend to accumulate on the skin surface and chorion, causing abnormality in the yolk sacs and pericardial edema [74]. Therefore, it is crucial to evaluate the toxicity of plant-mediated CuO NPs in various animal models.

## 6. Comparison of the Efficacy of Plant-Based-Synthesized and Commercial Cu and CuO NPs

Given the advantages of plant-based Cu/CuO NPs in the ease of production, as well as being eco-friendly, the efficacy of these NPs remains as one of the major concerns. Intriguingly, it was documented that the efficacy of these NPs in suppressing cancer proliferation was comparable to commercial CuO NPs or was even better in some of the cases (Table 6).

A detailed cytotoxic comparison study conducted by Rehana and her colleagues on green- and chemically synthesized CuO NPs revealed that green CuO NPs possessed higher toxicity against four different cancer cell lines. Among these, CuO NPs synthesized using *T. indica* showed more significant toxicity against human breast (MCF-7; IC_50_: 19.77 ± 0.98), cervical (HeLa; IC_50_: 20.32 ± 1.16), and lung (A549; IC_50_: 18.11 ± 0.93) cancer cells compared to commercially synthesized CuO-NP-treated human breast (MCF-7; IC_50_: 27.44 ± 2.14), cervical (HeLa; IC_50_: 45.31 ± 2.44), and lung (A549; IC_50_: 37.19 ± 2.82) cancer cells [54].

Despite inducing cytotoxicity in cancer cells, plant-mediated CuO NPs have less toxicity in healthy animals. In the zebrafish model, green-synthesized and commercial CuO NPs reduced the viability rate in a dose- and time-dependent manner [57]. However, the lethal dose (LC_50_) for green-synthesized CuO NPs was 175 ± 10 mg/L compared to 45 ± 10 mg/L for commercial CuO NPs [57]. More apoptotic cells were also found in zebrafish treated with commercial CuO NPs than zebrafish treated with green-synthesized CuO NPs [57]. These findings imply the potential of plant-based CuO NPs in cancer therapy without causing significant toxicity to normal cells.

Comparison of the effectiveness of these NPs in wound healing has been documented as well. It was evident that green CuO NPs have great wound healing properties compared to commercial wound ointments [71]. Compared to the control (2.5 ± 0.2 cm^2^) wound area, the green-CuO-NP-treated group decreased the area to 0.9 ± 0.2 cm^2^, whereas the CuSO_4_ ointment group decreased the area to 2.1 ± 0.1 cm^2^, suggesting green CuO NPs as a better candidate in promoting wound healing [71].

## 7. Current Status of Cu and CuO NPs and Their Future Perspective in Cancer Therapy

In recent years, NPs have been used as nanomedicines that can be utilized as delivery agents by encapsulating drugs or attaching therapeutic drugs and delivering them to the targeted tissues or cells more effectively [126,127]. They are designed in very small sizes to allow free movement in the human body to target cancer cells [128]. The Food and Drug Administration (FDA) has approved some NPs, such as Au, Ag, gadolinium oxide, iron oxide, nickel oxide, zinc oxide, and silica NPs, for biomedical applications to cure chronic diseases and treat cancers [128,129]. These NPs are used to treat liver, breast, cervical, and lung carcinoma cancers [129].

The choice of plant materials in the green synthesis of Cu and CuO NPs should be based on scientific evidence. Although plants naturally contain phytochemicals that act as reducing, stabilizing, and capping agents, different plants have different contents. This clearly shows that a strong scientific background study on the taxonomy of plants is needed to understand the principle and function of these phytochemicals in green synthesis [130]. From Table 1, most of the studies have suggested that common active compounds, such as polyphenols and flavonoids, play crucial roles in green synthesis. However, other compounds, especially alkaloids, consist of more than 12,000 different cyclic nitrogen compounds that also coordinate the green synthesis process [131].

Fierascu et al. (2020) stated that research output on the green synthesis of metallic NPs has increased gradually every year; however, it is mostly focused on antimicrobials and less exploration in anticancer studies [132]. Although extensive in vitro studies have demonstrated the therapeutic potential of plant-derived synthesis of Cu and CuO NPs, these NPs are still far from clinical trials due to the limitation on in vivo data. Further in-depth investigation of the toxicology and pharmacokinetics of these NPs is essential prior to clinical study. Additionally, the underlying mechanisms of Cu/CuO NPs in different disease models need to be evaluated. The optimal size and morphology of NPs need to be taken into consideration for the best uptake. Despite these challenges, the current encouraging data suggest the potential of Cu and CuO NPs as nanomedicine for cancer therapy and other diseases in the future.

## 8. Conclusions

Advancement in the green synthesis of NPs has created a new approach in biomedical applications. Plant-synthesized Cu and CuO NPs have many attractive properties, such as antibacterial, antifungal, anticancer, anti-inflammatory, and wound healing. It is important to note that the Cu/CuO NPs synthesized from different plants have different properties due to the diversity in metabolite composition. This diversity leads to variation in size, shape, and morphology of the NPs, which contributed to the alteration of their overall properties and activities. Additionally, further validation of the underlying mechanisms and pathways of Cu/CuO NPs at both the cellular and organism level is mandatory. Therefore, a comprehensive study on these NPs, including in vitro and in vivo assessments from different aspects, would drive their medicinal application. Although it seems that many trials and more research need to be conducted, the potential and prospects for developing Cu and CuO NPs as a future drug, especially in cancer therapy, are still very bright. The green technology of NPs, utilizing natural plant resources that will be developed into nanobiotechnology, is not very far off from a breakthrough.

## Figures and Tables

**Table 1:** Summary of plant-derived Cu and CuO nanoparticles biosynthesized.

Plant Used (Common Name)	Parts of Plant	Plant Metabolites Involved in Bioreduction	Precursor	Tmp. (°C)	pH	Time of Reaction	Cu/CuO NPs		Reference
							Size (nm)	Shapes	
*Abies spectabilis* (East Himalayan fir)	Leaves	Terpenoids, flavonoids, lignans, steroids, and phenols	CuSO_4_	27	nil	2 h	50	Spherical	[45]
*Abutilon indicum* (Indian mallow)	Leaves	Phenols and flavonoid	Cu(NO_3_)_2_·3H_2_O	400 ± 5 (Burned)	nil	2–5 min	16.78	Spherical	[46]
*Ailanthus altissima* (Varnish tree)	Leaves	Proteins, phenols, and alkenes	Cu(OAc)_2_	27	nil	4 h	5–20	Spherical	[47]
*Alchornea cordifolia* (Christmas Bush)	Leaves	Phenols, steroids, tannins, alkaloids, flavonoids, and xanthones	CuSO_4_·5H_2_O	80–90 °C	nil	4 h	16.25	Spherical	[48]
*Allium eriophyllum* Boiss (Kurdish traditional medicine plant)	Leaves	Neophytadiene and stigmast-5-en-3-ol	CuSO_4_	80	nil	16 h	30–35	Spherical	[49]
*Allium saralicum*	Leaves	Linolenic acid and methyl ester	CuSO_4_	nil	12	>1 h	45–50	Spherical	[50]
*Allium sativum* (Garlic)	Bulb	Polypenols and saponin	Cu(NO_3_)_2_	70	nil	2–3 h	20–40	Spherical and oval-shaped	[51]
*Annona muricata* (Soursop)	Leaves	Flavonoids and phenols	CuSO_4_·5H_2_O	80	12	nil	30–40	Spherical and cubical	[52]
*Azadirachta indica* (Neem tree)	Leaves	Phenols, flavonoids, carbohydrate, and saponin	CuSO_4_	27	nil	nil	36 ± 8	Spherical	[53]
*Azadirachta indica* (Neem tree)	Leaves	Phenols and flavonoids	Cu(OAc)_2_·4H_2_O	80	nil	nil	12	Spherical	[54]
*Brassica oleracea var acephala* (Kale)	leaves	Flavanoids, tannins, terpenoids, and phytosterols	CuSO_4_	27	nil	15 min	60–100	Spherical	[55]
*Beta vulgaris* (Beet)	Leaves	Alcohol and phenol	CuSO_4_·5H_2_O	60	nil	30 h	11.4–63.9	Spherical and irregular	[56]
*Calotropis gigantea* (Crown flower)	Floral	Flavonol glycosides, cardenolides, saccharides, and lipids	CuCl_2_	37	nil	24 h	25–35	Spherical	[57]
*Calotropis gigantean* (Crown flower)	Floral	Polysaccharides, proteins, and lipids	CuCl_2_	37	nil	24 h	32 ± 0.9	Spherical	[58]
*Camellia sinensis* (Black tea)	Leaves	Polyphenols and epigallocatechin gallate	Cu(NO_3_)_2_·3H_2_O	75	nil	12 h	22–39	Spherical	[59]
*Camellia sinensis* (Green tea)	Leaves	Polyphenols	CuSO_4_·5H_2_O	95	nil	nil	67–99	Spherical	[60]
*Camellia sinensis* (Green tea)	Leaves	Polyphenols	CuCl_2_·2H_2_O	90	nil	nil	10–40	Spherical	[37]
*Cissus arnottiana*	Leaves	Biomolecules	CuSO_4_	27	nil	4 h	60–90	Spherical	[61]
*Cissus vitiginea* (South Indian treebine)	Leaves	Polyphenol, anthroquinone, steroids, terpenoids, and tannins	CuSO_4_	27	nil	nil	5–20	Spherical	[62]
*Citrus* (Orange, lemon, tangerine)	Peel of fruit	Phenol	Cu(NO_3_)_2_⋅5H_2_O	80	nil	1 h	48–76	Globular	[63]
*Cordia sebestena* (Geiger tree)	Floral	Polyphenols, flavonoids, and tannins	Cu(NO_3_)_2_⋅3H_2_O	80	nil	4 h	20–35	Spherical	[64]
*Eclipta prostrate* (False daisy)	Leaves	Steroids, triterpenes, and flavonoids,	Cu(OAc)_2_	50	6	30 min	23–57	Spherical	[65]
*Eucalyptus globulus* (Southern blue gum)	Leaves	Phenol, terpenoids, flavonoids, and tannins	CuSO_4_	30–140	8	2–6 h	12–68	Cuboidal, spherical, and oval-shaped	[66]
*Euphorbia pulcherrima* (Poinsettia)	Floral	Flavonoids and amino acids	Cu(OAc)_2_·H_2_O	27	4	nil	16.3–153.7	Cubical	[67]
*Falcaria vulgaris* (Sickleweed; longleaf)	Leaves	Carvacrol and spathulenol	CuSO_4_	nil	12	>1 h	20	Spherical	[68]
*Ficus religiosa* (Sacred fig)	Leaves	Alkaloids, flavonoids, and terpenoids	CuSO_4_·5H_2_O	27	nil	nil	577	Spherical	[69]
*Ficus religiosa* (Sacred fig)	Leaves	Alkaloids, flavonoids, and terpenoids	CuSO_4_	27	nil	nil	577	Spherical	[70]
*Fragaria ananassa* (Strawberry)	Fruit	Flavonol, tannins, and anthocyanins	CuSO_4_	27	8	1 h	10–30	Spherical	[71]
*Hibiscus rosa-sinensis* (Chinese hibiscus)	Leaves	Phenols and flavonoids	Cu(OAc)_2_·4H_2_O	80	nil	nil	12	Spherical	[54]
*Ixoro coccinea* (Jungle geranium)	Leaves	Phenols and alcohols	CuSO_4_·5H_2_O	27	nil	24 h	80–110	Spherical	[72]
*Lawsonia inermis* (Henna)	Leaves	Hennotannic acid (naphthoquinone), mannitol, and alkaloids	Cu(NO_3_)_2_⋅3H_2_O	80	nil	12 h	22–38	Spherical	[73]
*Magnolia champaca* (Champak)	Floral	Starch, flavanol glycosides, and phenol	Cu(OAc)_2_	37	nil	24 h	20–40	Spherical	[74]
*Manilkara zapota* (Sapodilla)	Leaves	Triterpenoids, flavonoid glycosides, and polyphenol	CuSO_4_·5H_2_O	100	12	Until color change to brownish-black	18.9–45.2	Spherical	[75]
*Millettia pinnata* (Seashore Mempari; Pongam)	Flower	Proteins, acids, flavonoids, polyphenols, carboxylic acid, and alkaloids	Cu_2_(OAc)_4_(H_2_O)_2_	25 and 60	nil	nil	23 ± 1.10	Spherical	[76]
*Moringa oleifera* (Drumstick tree)	Leaves	Phenols and flavonoids	Cu(OAc)_2_·4H_2_O	80	nil	nil	12	Spherical	[54]
*Murraya koenigii* (Curry tree)	Leaves	Phenols and flavonoids	Cu(OAc)_2_·4H_2_O	80	nil	nil	12	Spherical	[54]
*Nilgirianthus ciliates* (Sahachara)	Leaves	Phenol, sapanonin, and tannins	CuSO_4_·5H_2_O	100	nil	30 min	20	Spherical	[77]
*Olea europaea* (Olive)	Leaves	Flavonoids	CuSO_4_·5H_2_O	100	nil	24 h	20–50	Spherical	[78]
*Phaseolus vulgaris* (Common bean)	Fruits	Phenolic, protease inhibitors, phytic acids, and saponins	CuSO_4_·5H_2_O	120	nil	7–8 h	26.6	Spherical	[79]
*Phoenix dactylifera L.* (Date palm)	Leaves	Polyphenols, flavonoids, and tannins	CuSO_4_·5H_2_O	70	nil	2 h	20–28	Spherical	[80]
*Pterolobium hexapetalum* (Indian redwing)	Leaves	Phenols, flavonoid, terpenoids, tannins, alkaloids, carbohydrates, and glycosides	CuSO_4_·5H_2_O	60	nil	2 h	10–50	Spherical	[81]
*Saccharum officinarum* (Sugarcane)	Stem	Glucose, sucrose, and fructose	Cu(NO_3_)_2_	80	10	9 h	29.5–60.5	Spherical, square, cube, plate, rectangular	[82]
*Stachys lavandulifolia* (Tea)	Leaves	Biomolecules	CuCl_2_	50	10	nil	<80	Spherical	[83]
*Syzygium alternifolium* (Mogi)	Fruit	Phenol and primary amines of protein	CuSO_4_·5H_2_O	50	8.2– 9	2 h	2–69	Spherical	[84]
*Tamarindus indica* (Tamarinda; Asam jawa)	Leaves	Phenols and flavonoids	Cu(OAc)_2_·4H_2_O	80	nil	nil	12	Spherical	[54]
*Terminalia bellirica* (Bahera)	Fruits	Tannins	Cu(NO_3_)_2_	25	nil	nil	9–14	Spherical	[85]
*Tribulus terrestris* (Bindii)	Fruit	Alkaloids, flavonoids, tannins, ascorbic acid, and phenols	CuSO_4_·5H_2_O	90	nil	2 h	5–22	Spherical	[86]
*Tridax procumbens* (Tridax daisy)	Leaves	Hexadecen, pentadecne, and squalene	CuSO_4_	80	nil	4 h	16	Spherical	[87]

**Table 2:** Antibacterial effects of plant-biosynthesized Cu and CuO NPs.

Bacterial Species	Cu/CuO NPs			Diameter of Inhibition Zone (mm)/Inhibition (%)	Reference
	Size (nm)	Shapes	Concentration/ Amount		
**Gram-negative**					
*Campylobacter coli*	48–76	Globular	25 μg/mL	20 (orange peel extract)	[63]
				16 (lemon peel extract)	
			50 μg/mL	26 (orange peel extract)	
				25 (lemon peel extract)	
*Escherichia coli*	5–20	Spherical	100 μg/mL	18	[47]
	5–22	Spherical	MIC: 16 μg/mL	–	[86]
	10–30	Spherical	4 mg/mL	12.4 ± 1.3	[71]
	10–50	Spherical	50 μg/mL	14 ± 0.22	[81]
	16.8	Spherical	3 mg	6 ± 0.09	[46]
			5 mg	7 ± 0.08	
	18.9–45.2	Spherical	5 μg/mL	98%	[75]
	20	Spherical	1000 μg/mL	13	[77]
	20–40	Spherical and oval-shaped	50 μg/mL	3.90 ± 0.27	[51]
			100 μg/mL	8.80 ± 0.54	
			150 μg/mL	11.65 ± 0.67	
	29.5–60.5	Spherical, square, cube, plate, and rectangular	100 μg	5	[82]
	30–35	Spherical	4 mg/mL	14.2 ± 0.83	[49]
	48–76	Globular	25 μg/mL	18 (orange peel extract)	[63]
			50 μg/mL	24 (orange peel extract)	
	60–100	Spherical	25 μL	24	[55]
			50 μL	32	
	67–99	Spherical	10 μL of 170 mL of 1 mM CuSO_4_·5H_2_O aqueous solution + 30 mL of 1% green tea extract	24 ± 1.73	[60]
*Klebsiella*	16.8	Spherical	3 mg	12 ± 0.04	[46]
			5 mg	14 ± 0.05	
*Klebsiella pneumonia*	20–40	Spherical and oval-shaped	50 μg/mL	3.50 ± 0.24	[51]
			100 μg/mL	8.55 ± 0.52	
			150 μg/mL	10.65 ± 0.63	
*Moraxwlla catarrhalis*	48–76	Globular	25 μg/mL	18 (orange peel extract)	[63]
			50 μg/mL	24 (orange peel extract)	
*Proteus mirabilis*	10–30	Spherical	8 mg/mL	13.2 ± 1.3	[71]
*Pseudomonas aeruginosa*	5–22	Spherical	MIC: 17.5 μg/mL	–	[86]
	10–30	Spherical	4 mg/mL	13.8 ± 0.4	[71]
	20	Spherical	1000 μg/mL	17	[77]
	20–40	Spherical and oval-shaped	50 μg/mL	3.75±0.26	[51]
			100 μg/mL	8.60 ± 0.53	
			150 μg/mL	10.90 ± 0.64	
	29.5–60.5	Spherical, square, cube, plate, and rectangular	100 μg	8	[82]
	30–35	Spherical	2 mg/mL	13.2 ± 0.44	[49]
	60–100	Spherical	25 μL	16	[55]
			50 μL	31	
*Salmonella typhi*	67–99	Spherical	10 μL of 170 mL of 1 mM CuSO_4_·5H_2_O aqueous solution + 30 mL of 1% green tea extract	21 ± 1.00	[60]
*Salmonella typhimurium*	10–30	Spherical	8 mg/mL	16.8 ± 1	[71]
	30–35	Spherical	4 mg/mL	12 ± 0	[49]
*Vibrio harveyi*	18.9–45.2	Spherical	5 μg/mL	98%	[75]
*Vibrio parahaemolyticus*	18.9–45.2	Spherical	5 μg/mL	98%	[75]
**Gram-positive**					
*Bacillus cereus*	5–22	Spherical	MIC: 21 μg/mL	–	[86]
*Bacillus subtilis*	10–30	Spherical	4 mg/mL	14.6 ± 0.8	[71]
	10–50	Spherical	50 μg/mL	15 ± 0.29	[81]
	16.8	Spherical	3 mg	15 ± 0.07	[46]
			5 mg	15 ± 0.11	
	18.9–45.2	Spherical	5 μg/mL	50%	[75]
	20–40	Spherical and oval-shaped	50 μg/mL	3.35 ± 0.23	[51]
			100 μg/mL	8.20 ± 0.50	
			150 μg/mL	10.90 ± 0.62	
	29.5–60.5	Spherical, square, cube, plate, and rectangular	100 μg	9	[82]
	30–35	Spherical	2 mg/mL	13.2 ± 0.83	[49]
*Clostridium perfringens*	48–76	Globular	25 μg/mL	12 (orange peel extract)	[63]
			50 μg/mL	19 (orange peel extract)	
*Listeria monocytogenes*	48–76	Globular	25 μg/mL	9 (lemon peel extract)	[63]
			50 μg/mL	13 (lemon peel extract)	
*Micrococcus luteus*	67–99	Spherical	10 μL of 170 mL of 1 mM CuSO_4_·5H_2_O aqueous solution + 30 mL of 1% green tea extract	23.33 ± 2.08	[60]
*Staphylococcus aureus*	5–20	Spherical	80 μg/mL	20	[47]
	5–22	Spherical	MIC: 19.5 μg/mL	–	[86]
	10–30	Spherical	4 mg/mL	12.6 ± 0.8	[71]
	10–50	Spherical	50 μg/mL	15 ± 0.47	[81]
	16.8	Spherical	3mg	6 ± 0.09	[46]
			5 mg	10 ± 0.11	
	18.9–45.2	Spherical	5 μg/mL	> 90%	[75]
	20	Spherical	1000 μg/mL	15	[77]
	20–40	Spherical and oval-shaped	50 μg/mL	2.80 ± 0.19	[51]
			100 μg/mL	7.50 ± 0.45	
			150 μg/mL	11.30 ± 0.58	
	29.5–60.5	Spherical, square, cube, plate, and rectangular	100 μg	9	[82]
	30–35	Spherical	2 mg/mL	15.4 ± 1.34	[49]
	48–76	Globular	25 μg/mL	13 (orange peel extract)	[63]
				17 (lemon peel extract)	
			50 μg/mL	25 (orange peel extract)	
				23 (lemon peel extract)	
	60–100	Spherical	25 μL	14	[55]
			50 μL	24	
*Staphylococcus saprophyticus*	10–30	Spherical	2 mg/mL	12.4 ± 0.5	[71]
*Streptococcus mutans*	20	Spherical	1000 μg/mL	13	[77]
	67–99	Spherical	10 μL of 170 mL of 1 mM CuSO_4_·5H_2_O aqueous solution + 30 mL of 1% green tea extract	30 ± 2.00	[60]
*Streptococcus pneumonia*	10–30	Spherical	4 mg/mL	11.8 ± 1	[71]
	30–35	Spherical	2 mg/mL	15.2 ± 0.83	[49]
	48–76	Globular	25	8 (tangerine peel extract)	[63]
			50	14 (tangerine peel extract)	
*Streptococcus pyrogenes*	20–40	Spherical and oval-shaped	50 μg/mL	3.05 ± 0.21	[51]
			100 μg/mL	8.15 ± 0.50	
			150 μg/mL	10.65 ± 0.60	

MIC: minimal inhibitory concentration.

**Table 3:** Antifungal effects of plant-biosynthesized Cu and CuO NPs.

Fungal Species	CuO NPs			Diameter of Inhibition Zone (mm)/INHIBITION (%)	Reference
	Size (nm)	Shapes	Concentration		
*Aspergillus flavus*	5–24	Cuboidal, spherical, oval-shaped	10 μg/mL	9.0 ± 0.13	[66]
			25 μg/mL	13.16 ± 0.49	
			50 μg/mL	16.9 ± 0.42	
	20–40	Spherical, oval-shaped	50 μg/mL	2.35 ± 0.16	[51]
			100 μg/mL	6.25 ± 0.36	
			150 μg/mL	9.30 ± 0.58	
*Aspergillus fumigates*	20–40	Spherical, oval-shaped	50 μg/mL	2.70 ± 0.18	[51]
			100 μg/mL	7.00 ± 0.42	
			150 μg/mL	9.95 ± 0.65	
*Aspergillus niger*	20–40	Spherical, oval-shaped	50 μg/mL	2.70 ± 0.18	[51]
			100 μg/mL	6.60 ± 0.39	
			150 μg/mL	9.96 ± 0.61	
*Candida albicans*	10–30	Spherical	4 mg/mL	12 ± 1.2	[71]
	20–40	Spherical, oval-shaped	50 μg/mL	2.95 ± 0.20	[51]
			100 μg/mL	6.85 ± 0.40	
			150 μg/mL	10.05 ± 0.63	
	30–35	Spherical	4 mg/mL	9.0 ± 1.22	[49]
	60–100	Spherical	25 μL	18	[55]
			50 μL	21	[55]
*Candida glabrata*	10–30	Spherical	4 mg/mL	13 ± 1	[71]
	30–35	Spherical	4 mg/mL	10.2 ± 0.83	[49]
*Candida guilliermondii*	10–30	Spherical	2 mg/mL	13.8 ± 1	[71]
	30–35	Spherical	2 mg/mL	8.6 ± 0.89	[49]
*Candida krusei*	10–30	Spherical	2 mg/mL	12.4 ± 0.5	[71]
	30–35	Spherical	2 mg/mL	10.2 ± 0.83	[49]
*Candida parapsilosis*	10–30	Spherical	2 mg/mL	14.8 ± 1	[71]
*Rhizoctonia solani*	18.9–45.2	Spherical	50 μg/mL	24.4%	[75]
			100 μg/mL	56.6%	
			200 μg/mL	65.5%	
*Sclerotium oryzae*	18.9–45.2	Spherical	50 μg/mL	61.1%	[75]
			100 μg/mL	88.9%	
			200 μg/mL	100%	

**Table 4:** Anticancer effects of the plant-derived Cu and CuO NPs biosynthesized.

Types of Cells/ Cell Line	Cu/CuO NPs		Toxicity (IC_50_) (μg/mL)	Biological Function (Targeting)	Reference
	Size (nm)	Shapes			
**Breast cancer**					
AMJ-13	20–50	Spherical	1.47	Antioxidant, loss of membrane potential, and DNA fragmentation	[78]
MCF-7	5–24	Cuboidal, spherical, and oval-shaped	>100	ROS generation, loss of mitochondrial membrane potential, apoptosis, and cell cycle arrest	[66]
	10–40	Spherical	50.3	Growth inhibition	[37]
	12	Spherical	19.77–27.44 (depends on source of plant extract)	Antioxidant and apoptosis	[54]
	18.9–45.2	Spherical	53.89	Growth inhibition	[75]
	20	Spherical	85.58	Growth inhibition	[77]
	20	Spherical	24.5	ROS generation and antiangiogenic	[111]
	30–40	Spherical, cubical	35	Growth inhibition	[52]
	36 ± 8	Spherical	21.56	ROS and NO generation, apoptosis, DNA fragmentation, induces proinflammatory (TNF-α) cytokines, and inhibits anti-inflammatory cytokine (IL-10)	[53]
	>200	Spherical	21.5	ROS generation and antiangiogenic	[111]
MDA-MB-231	10–50	Spherical	30	ROS generation	[81]
	20	Spherical	11	ROS generation and antiangiogenic	[111]
	>200	Spherical	7.5	ROS generation and antiangiogenic	[111]
**Cervical cancer**					
HeLa	12	Spherical	26.73–20.32 (depends on the source of plant extract)	Antioxidant and apoptosis	[54]
	~26.6	Spherical	~0.5 mg/mL	ROS generation, loss of mitochondrial membrane potential, and apoptosis	[79]
	36 ± 8	Spherical	24.74	ROS and NO generation, apoptosis, DNA fragmentation, induces proinflammatory (TNF-α) cytokines, and inhibits anti-inflammatory cytokine (IL-10)	[53]
	60–100	Spherical	119.1 μg/mL	Growth inhibition	[55]
**Colon cancer**					
HT-29	10–40	Spherical	33.0	Growth inhibition	[37]
**Epithelioma**					
Hep-2	12	Spherical	21.66–29.58 (depends on source of plant extract)	Antioxidant and apoptosis	[54]
**Gastric cancer**					
AGS	5–22	Spherical	25–50	Apoptosis	[86]
**Leukemia**					
MOLT-4	10–40	Spherical	>80	Growth inhibition	[37]
**Liver cancer**					
HepG2	23–57	Spherical, hexagonal, cubical	>500	Antioxidant	[65]
**Lung cancer**					
A549	12	Spherical	18.11–37.19 (depends on the source of plant extract)	Antioxidant and apoptosis	[54]
	20	Spherical	81.57	Growth inhibition	[77]
	33.47	Spherical, irregular	25	Apoptosis	[56]
	577	Spherical	200	Loss of mitochondrial membrane potential, ROS generation, and apoptosis	[69]
	577	Spherical	200	Regulates histone deacetylases, downregulates oncogenes and upregulates tumor suppressor genes, intrinsic and extrinsic apoptosis, and downregulates inflammatory genes (TNF-α and COX-2)	[70]
**Ovarian cancer**					
SKOV-3	20–50	Spherical	2.27	Antioxidant, loss of membrane potential, and DNA fragmentation	[78]

ROS, reactive oxygen species; NO, nitric oxide; TNF-α, tumor necrosis factor; IL-10, interleukin-10.

**Table 5:** Effects of the plant-derived Cu and CuO NPs biosynthesized on cell lines and animal models.

Types of Cells/ Cell Line/Animal	Cu/CuO NPs		Toxicity (IC_50_)	Reference
	Size (nm)	Shapes		
**Human embryonic kidney cells**				
HEK 293	32 ± 0.9	Spherical	410 μg/mL	[58]
**Human umbilical vein endothelial cells**				
HUVEC	10–30	Spherical	>1000 μg/mL	[71]
**Human dermal fibroblast**				
NHDF	12	Spherical	>100 μg/mL	[54]
HuFb	20–50	Spherical	54.34 μg/mL	[78]
L929	20	Spherical	>100 μg/mL	[77]
**Animal**				
Male Swiss albino mice (BALB/c strain)	20–50	Spherical	Lethal at 800 mg/kg	[78]
Zebrafish	20–40	Spherical	500 ± 15 mg/L	[74]

**Table 6:** Comparison of the toxicity of plant-derived and commercialized CuO NPs.

Cell Line/ Animal Model	Plant-Mediated Cu/CuO NPs			Commercial Cu/CuO NPs			Reference
	Size (nm)	Shapes	Toxicity/ Area	Size (nm)	Shapes	Toxicity/ Area	
**In vitro**							
MCF-7	12	Spherical	19.77 ± 0.98 μg/mL	12	Spherical	27.44 ± 2.14 μg/mL	[54]
HeLa	12	Spherical	20.32 ± 1.16 μg/mL	12	Spherical	45.31 ± 2.44 μg/mL	[54]
A549	12	Spherical	18.11 ± 0.93 μg/mL	12	Spherical	37.19 ± 2.82 μg/mL	[54]
**In vivo**							
Zebrafish	25–35	Spherical	175 ± 10 mg/L	25–35	Spherical	45 ± 10 mg/L	[57]
Rat	10–30	Spherical	Wound area: 0.9 ± 0.2 cm^2^	10–30	Spherical	Wound area: 2.1 ± 0.1 cm^2^	[71]

## Data Availability

Not applicable.
